# Predicting Activities of Daily Living (ADL) Outcomes in Recovery-Phase Stroke Patients Using the Trunk Impairment Scale: A Validation Study

**DOI:** 10.7759/cureus.96450

**Published:** 2025-11-09

**Authors:** Masahiro Ishiwatari, Akihiro Ogawa, Satoshi Hakukawa, Kazuya Fukae, Masayuki Sugo, Ryo Abe, Masashi Zenta, Satoshi Kido

**Affiliations:** 1 Faculty of Health Sciences, Department of Physical Therapy, Uekusa Gakuen University, Chiba, JPN; 2 Faculty of Social Work Studies, Department of Physical Therapy, Josai International University, Chiba, JPN; 3 Rehabilitation, Reiwa Rehabilitation Hospital, Chiba, JPN; 4 Department of Rehabilitation and Care, Kiminomori Rehabilitation Hospital, Chiba, JPN; 5 Graduate Course of Health and Social Services, Saitama Prefectural University, Saitama, JPN

**Keywords:** functional independence measure, outcome prediction, stroke rehabilitation, subacute stroke, trunk impairment scale

## Abstract

Trunk function is a key determinant of activities of daily living (ADL) after stroke. While the Trunk Impairment Scale (TIS) has been linked to functional outcomes, its prognostic utility in the recovery phase is less established. This study aimed to develop and validate an ADL prediction model at discharge using the TIS in recovery-phase stroke patients. This prospective cohort study included 80 first-ever stroke patients admitted to Kiminomori Rehabilitation Hospital in Chiba, Japan. Trunk function (TIS), motor function (Stroke Impairment Assessment Set (SIAS)-M), stroke severity (NIH Stroke Scale/Score (NIHSS)), and ADL ( Functional Independence Measure (FIM)-M) were assessed. Three regression models were compared, and predictive validity was tested using cross-validation and bootstrap analysis. The TIS model showed the highest predictive accuracy, outperforming baseline and motor function models. Bootstrap analysis confirmed the independent contribution of one-month TIS to discharge ADL outcomes. The TIS assessed one month after stroke is a reliable predictor of discharge ADL, supporting its use for individualized rehabilitation planning.

## Introduction

Trunk function is a key determinant of activities of daily living (ADL) after stroke, as it provides the foundation for postural control and mobility [1-3]. Trunk function, defined as the integrated capacity for coordinated movement, proprioceptive regulation, and trunk muscular strength, plays a key role in supporting postural stability and efficient functional activity after stroke. Impairment of trunk stability can compromise independence, while targeted trunk training has been shown to improve balance, gait, and quality of life [4-5]. Prognostic prediction after stroke depends on multiple factors, among which trunk function plays a particularly critical role [6-7].

In recent years, shorter hospital stays and the need for seamless transition to recovery-phase rehabilitation have increased the importance of reliable prognostic tools [8-11]. During this stage of heightened neuroplasticity [12-14], accurate prediction of functional recovery supports individualized rehabilitation planning and discharge preparation [6,10].

Trunk control is especially important for basic motor tasks, wheelchair-level ADL, and reducing caregiver burden [15-18]. Several assessment tools have been developed, but many focus only on sitting balance or isolated abilities [19-20]. The Trunk Impairment Scale (TIS) overcomes these limitations by evaluating both trunk balance and functional components [21]. While its association with ADL has been reported, evidence for its predictive utility in the recovery phase remains limited.

Recent findings have also demonstrated that early TIS scores can predict gait independence after acute stroke [22], further supporting its potential as a prognostic indicator.

Therefore, the aim of this study was to develop and validate a prediction model for discharge ADL using the TIS in recovery-phase stroke patients. Such a model may enhance prognostic accuracy and support the design of individualized rehabilitation programs.

## Materials and methods

Participants

This prospective cohort study initially included 116 patients who were transferred from acute care hospitals to recovery-phase rehabilitation hospitals (Kiminomori Rehabilitation Hospital, Chiba, Japan) with a diagnosis of cerebral infarction or cerebral hemorrhage between December 2021 and March 2023.

The inclusion criterion was a first-ever unilateral cerebral infarction. At one month after admission to the recovery-phase rehabilitation ward, participants were required to have a level of consciousness classified as awake without stimulation (Glasgow Coma Scale ≥14).

Exclusion criteria included impaired consciousness (Glasgow Coma Scale ≤14), surgical intervention, stroke deterioration, or death. Stroke deterioration was defined as an increase of ≥4 points in the National Institutes of Health Stroke Scale (NIHSS) score between admission and one month after admission to the recovery-phase ward.

Based on these criteria, the final study population consisted of 80 participants (46 men and 34 women).

All participants received a detailed explanation of the study’s purpose, and written informed consent was obtained. For patients unable to provide a signature, consent was obtained from an authorized representative, such as a family member. The study was approved by the Ethics Committee of Kiminomori Rehabilitation Hospital, Chiba, Japan (approval no. 2021-11), and all procedures were conducted in accordance with the principles of the Declaration of Helsinki.

Methods

Demographic data, including age, sex, and length of stay (LOS), were extracted from electronic medical records. Trunk function was assessed using the 7-item Trunk Impairment Scale (TIS; Fujiwara version) [21]. The scale consists of seven performance-based items, with a total score ranging from 0 to 21 points, where higher scores indicate better trunk function.

Two items are adapted from the Stroke Impairment Assessment Set (SIAS) and assess abdominal muscle strength and postural verticality, while the remaining five items uniquely evaluate trunk verticality perception, rotational trunk strength on both the affected and unaffected sides, and bilateral righting reactions.

The Fujiwara version of the TIS has demonstrated high reliability and validity and is widely used in clinical and research settings to quantify trunk impairment after stroke. Stroke severity was assessed using the National Institutes of Health Stroke Scale (NIHSS) [23]. Motor function on the affected side was evaluated using the motor items of the SIAS (SIAS-M) [24], and ADL were assessed with the motor subscale of the Functional Independence Measure (FIM-M) [25].

Only total scores were analyzed, without reproducing or describing any individual items, in accordance with copyright restrictions.

Although both the motor and cognitive subscales of the FIM were collected, only the motor subscale (FIM-M) was used in the analysis because the study focused on physical ADL performance in relation to trunk function, and the discharge FIM was assessed uniformly on the day before discharge for all participants.

Written confirmations regarding appropriate usage conditions were obtained from Wolters Kluwer (TIS and NIHSS), SAGE Publications (SIAS), and UDSMR/Netsmart (FIM).

To account for variability in physical function, assessments were performed one month after admission and again at discharge.

All evaluations were conducted by the same examiner.

Statistical analysis

The Shapiro-Wilk test was used to examine whether variables followed a normal distribution. To address multicollinearity, Spearman’s rank correlation coefficients were calculated, and variables with |r| ≥ 0.9 were excluded. Variance inflation factors (VIFs) ≥ 10 were also used to identify multicollinearity.

Multiple regression analyses were performed to evaluate the predictive validity of discharge ADL. The baseline model included age, length of stay, and one-month FIM-M as explanatory variables, with discharge FIM-M as the dependent variable. The TIS model additionally included one-month TIS, and the SIAS-M model included one-month SIAS-M. For each model, the coefficient of determination (R²), regression coefficients, and standard errors were calculated to compare predictive accuracy.

To assess generalizability, 10-fold cross-validation was performed, and R² and mean squared error (MSE) were calculated for each fold. In addition, bootstrap analysis (1,000 resamplings) was conducted to further validate model stability and reliability, estimating bias, standard errors, and 95% confidence intervals for regression coefficients and predictive accuracy.

All statistical analyses were conducted using IBM SPSS Statistics for Windows, version 29.0 (released 2022, IBM Corp., Armonk, NY) and R version 4.3.1 (R Foundation for Statistical Computing, Vienna, Austria). Statistical significance was set at p < 0.05.

## Results

Participant characteristics

Participant characteristics are summarized in Table 1. The median age of participants was 73 years (interquartile range (IQR): 64-80), and the median length of hospital stay was 139 days (IQR: 97-149). The cohort included 46 males and 34 females, with 41 patients presenting right-sided and 39 left-sided lesions. Stroke type consisted of 50 cerebral infarctions and 30 cerebral hemorrhages.

**Table 1:** Participant characteristics FIM-M: Functional Independence Measure, motor subscale; TIS: Trunk Impairment Scale; SIAS-M: Stroke Impairment Assessment Set, motor items

Characteristic			Value	
Number of patients (male/female)			80 (46/34)	
Age (years, median, IQR)			73 (64-80)	
Length of hospital stay (days, median, IQR)			139 (97-149)	
Side of lesion (right/left)			41/39	
Diagnosis				
Cerebral infarction			50	
Cerebral hemorrhage			30	
Outcome measure			1 month	At discharge
FIM-M			43 (24-62)	71 (45-83)
TIS			16 (13-17)	18 (16-19)
SIAS-M			13 (5-18)	15 (8-20)

Correlation analysis between variables

**Figure 1:**
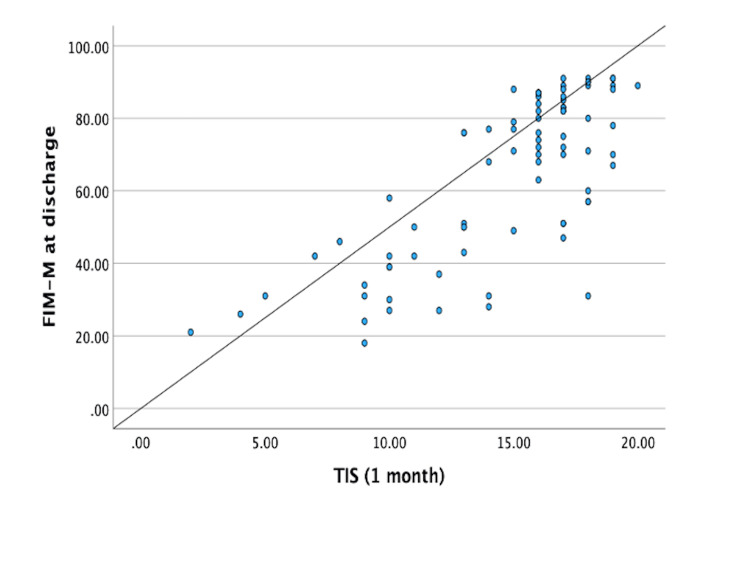
Scatter plot showing the correlation between one-month Trunk Impairment Scale (TIS) and discharge Functional Independence Measure motor score (FIM-M). A significant positive correlation was observed (Spearman’s ρ = 0.693, p < 0.001), indicating that higher TIS scores at one month were associated with better discharge FIM-M outcomes.

Correlation coefficients between variables are summarized in Table 2. A significant positive correlation was observed between discharge FIM-M and one-month TIS (Spearman’s ρ = 0.693, p < 0.001), indicating strong concurrent validity. This relationship is illustrated in Figure 1.

**Table 2:** Correlation coefficients between variables Spearman’s rank correlation coefficients are shown. **p < 0.01, **p < 0.001. FIM-M: Functional Independence Measure, motor subscale; TIS: Trunk Impairment Scale; SIAS-M: Stroke Impairment Assessment Set, motor items

	Age	Length of stay	FIM-M (one month)	TIS (one month)	SIAS-M (one month)	FIM-M at discharge
Age	1	-0.049	-0.319**	-0.203	-0.049	-0.480**
Length of stay	-0.049	1	-0.459**	-0.454**	-0.522**	-0.308**
FIM-M (one month)	-0.319**	-0.459**	1	0.749**	0.636**	0.795**
TIS (one month)	-0.203	-0.454**	0.749**	1	0.739**	0.693**
SIAS-M (one month)	-0.049	-0.522**	0.636**	0.739**	1	0.579**
FIM-M at discharge	-0.480**	-0.308**	0.795**	0.693**	0.579**	1

Multiple regression analysis

The results of the multiple regression analyses are presented in Table 3, which shows the contribution of each predictor to discharge FIM-M. In the baseline model, which included age, length of stay, and one-month FIM-M as independent variables with discharge FIM-M as the dependent variable, the adjusted R² was 0.68 (p < 0.001). When TIS was added, the adjusted R² increased to 0.74 (p < 0.001), indicating improved predictive validity. Similarly, when SIAS-M was added, the adjusted R² was 0.71 (p < 0.001).

**Table 3:** Multiple regression analysis predicting FIM-M at discharge FIM-M: Functional Independence Measure, motor subscale; TIS: Trunk Impairment Scale; SIAS-M: Stroke Impairment Assessment Set, motor items; B: unstandardized regression coefficient; SE: standard error; β: standardized coefficient

Dependent variable: FIM-M at discharge	Independent variable		Unstandardized coefficient		Standardized coefficient	t-value	P-value	Adjusted R²
			B	SE	β			
Model 1 (Baseline)	Constant		53.675	13.217		4.061	P＜0.001	0.68
	Length of stay		-0.003	0.043	-0.006	-0.078	0.938	
	Age		-0.347	0.113	-0.213	-3.062	0.003	
	FIM-M (one month)		0.809	0.087	0.736	9.267	P＜0.001	
Model 2 (TIS model)	Constant		31.983	12.838		2.491	0.015	0.74
	Length of stay		0.013	0.039	0.024	0.347	0.73	
	Age		-0.344	0.102	-0.211	-3.381	0.001	
	FIM-M (one month)		0.535	0.1	0.487	5.354	P＜0.001	
	TIS (one month)		2.126	0.482	0.366	4.409	P＜0.001	
Model 3 (SIAS-M model)	Constant		49.733	12.514		3.974	P＜0.001	0.71
	Length of stay		0.039	0.043	0.069	0.922	0.359	
	Age		-0.413	0.109	-0.253	-3.797	P＜0.001	
	FIM-M (one month)		0.639	0.097	0.582	6.566	P＜0.001	
	SIAS-M (one month)		0.893	0.275	0.277	3.254	0.002	

Cross-validation analysis

The results of the 10-fold cross-validation are shown in Table 4, which compares the predictive accuracy of the baseline, TIS, and SIAS-M models. For the baseline model, the mean adjusted R² was 0.516 and the mean squared error (MSE) was 169.76. For the TIS model, the mean adjusted R² improved to 0.587 and the MSE decreased to 141.63, demonstrating superior predictive accuracy compared with the baseline model. The SIAS-M model yielded a mean adjusted R² of 0.563 and an MSE of 152.98, which was better than the baseline model but not as strong as the TIS model.

**Table 4:** Results of the 10-fold cross-validation analysis Values represent mean adjusted R² and mean squared error (MSE) from 10-fold cross-validation. R²: coefficient of determination; MSE: mean squared error; TIS: Trunk Impairment Scale; SIAS-M: Stroke Impairment Assessment Set, motor items

Model	Mean adjusted R²	Mean squared error (MSE)
Baseline model	0.516	169.76
TIS model	0.587	141.63
SIAS-M model	0.563	152.98

Bootstrap analysis

The results of the bootstrap analysis (1,000 resamplings) are presented in Table 5, which displays the regression coefficients and confidence intervals for each predictor. The TIS model demonstrated the highest utility for predicting discharge FIM-M. In particular, one-month TIS contributed substantially and significantly to the dependent variable (β = 2.113, 95% CI: 0.965-3.212).

**Table 5:** Results of bootstrap analysis (1,000 resamplings) Values represent regression coefficients (β) with 95% confidence intervals (CI) obtained from 1,000 bootstrap resamplings. CI: confidence interval; TIS: Trunk Impairment Scale; FIM-M: Functional Independence Measure, motor items

Model	Variable	β	95% CI (lower – upper)
TIS model	TIS (1 month)	2.113	0.965 – 3.212

## Discussion

The aim of this study was to develop a prediction model for discharge ADL in recovery-phase stroke patients and to confirm its reliability and robustness. Multiple regression analysis identified associations among explanatory variables, and bootstrap methods verified predictive accuracy and confidence intervals. The results demonstrated that the TIS model provided superior predictive validity and reliability compared with the other models. Importantly, the one-month TIS score independently and substantially contributed to predicting discharge FIM-M. These findings underscore the clinical value of incorporating trunk function assessment into prognostic models and suggest that the TIS may serve as a useful tool for guiding individualized rehabilitation planning and discharge support.

In recent years, early rehabilitation has been increasingly emphasized, with evidence showing that very early intervention can significantly affect prognosis [13-14]. Given the shortened length of stay in acute care hospitals and the need for early transfer to recovery-phase facilities, identifying prognostic predictors is essential [8-9]. Previous studies have demonstrated that trunk control is a strong predictor of ADL outcomes at six months post-stroke [3,16], and measures such as the Fugl-Meyer motor score and the Brunnstrom Recovery Stage (BRS) have been reported as useful indicators for predicting motor and ADL outcomes at discharge [10-11]. However, assessments of trunk function have often focused mainly on sitting balance, which may not fully capture the multifaceted aspects of trunk control [20].

In this study, the baseline model incorporated admission FIM-M, while SIAS-M and TIS were added separately to examine their predictive contributions. The results showed that the TIS model outperformed both the baseline and SIAS-M models. Trunk stability and mobility are fundamental for ADL performance [17-18], and the TIS provides a comprehensive assessment including recognition of trunk verticality, righting reactions, and trunk rotational strength. Thus, the TIS can complement conventional balance assessments and provide a broader perspective for predicting ADL outcomes in recovery-phase stroke patients.

Our findings also indicate that TIS assessment at one month after onset is a reliable predictor of discharge FIM-M. Previous research has shown that TIS is strongly associated with trunk function, gait ability, and ADL and that assessment within 48 hours of onset predicts discharge FIM-M in the acute phase [1,22]. In our study, assessing TIS at one month -after transfer to recovery-phase rehabilitation - standardized the timing of evaluation and provided a fairer representation of progress. The adjusted R² of 0.74 confirmed the model’s predictive validity, suggesting that TIS retains prognostic value not only in the acute phase but also in the recovery phase.

Furthermore, bootstrap analysis confirmed the predictive accuracy and reproducibility of the TIS model, with one-month TIS showing a significant independent contribution (β = 2.113, 95% CI: 0.965-3.212). These results reinforce the novelty and clinical relevance of the present study. Because TIS evaluates both balance and functional performance, it offers valuable insights for designing rehabilitation programs and individualized interventions. At one month post-stroke, patients’ conditions are generally more stable than in the acute phase, and TIS scores may better reflect trunk function and rehabilitation progress.

Previous studies have supported the utility of TIS as a prognostic tool across different phases of stroke rehabilitation [1,21,22]. Recovery of trunk function from the acute to the chronic phase has been reported to influence long-term ADL performance [1,3,18]. Our study adds to this evidence by showing that TIS evaluation at one month improves the accuracy of predicting discharge outcomes. Collectively, these findings suggest that TIS is useful not only in the acute but also in the subacute stage and may contribute to long-term recovery planning.

This study has several limitations. Differences in non-training time and the duration or content of rehabilitation may have affected ADL improvements. In addition, because the study was conducted at a single rehabilitation hospital and included only Japanese patients, the generalizability of the findings may be limited. Although lesion territory and stroke type (ischemic vs. hemorrhagic) were documented for all participants, subgroup analyses were not performed because the sample size was insufficient for valid statistical comparisons. Given that these factors may influence motor recovery after stroke, future studies with larger cohorts are warranted to explore their potential impact on trunk function and ADL outcomes. In addition, although both motor and cognitive FIM subscales were collected, only FIM-M was analyzed because the study focused on physical ADL performance. Another limitation is that the follow-up period was restricted to the inpatient phase, and long-term outcomes such as community reintegration or quality of life were not examined. In addition, all assessments were performed by a single examiner, which may affect reproducibility. Future multicenter studies with larger and more diverse populations and longer follow-up periods are needed to confirm the external validity of the TIS and its relevance for long-term functional and social outcomes.

## Conclusions

This study demonstrated that the TIS, particularly when assessed one month after stroke onset, is a reliable predictor of discharge ADL outcomes in recovery-phase stroke patients. Compared with baseline and motor function models, the TIS model showed superior predictive accuracy and reproducibility, as confirmed by bootstrap validation. These findings suggest that the TIS is valuable not only in the acute phase but also in the subacute stage, offering clinicians a practical tool for individualized rehabilitation planning and discharge management. Future studies should evaluate long-term outcomes after community reintegration and examine the external validity of the TIS in relation to quality of life and social participation.
